# Lymphocyte to Monocyte Ratio: A New Independent Prognostic Factor in Bladder Cancer Progression?

**DOI:** 10.3389/fonc.2021.754649

**Published:** 2021-09-09

**Authors:** Matteo Ferro, Vincenzo Francesco Caputo, Biagio Barone, Ciro Imbimbo, Ottavio de Cobelli, Felice Crocetto

**Affiliations:** ^1^Department of Urology, IRCCS European Institute of Oncology (IEO), Milan, Italy; ^2^Department of Neurosciences, Reproductive Sciences and Odontostomatology, University of Naples Federico II, Naples, Italy

**Keywords:** lymphocyte to monocyte ratio (LMR), bladder cancer, prognostic factor, MIBC (muscle-invasive bladder cancer), NMIBC (non-muscle-invasive bladder cancer)

Bladder cancer (BC) is the fourth most common cancer in men and the eleventh most common cancer in women. Characterized by significant morbidity and mortality, BC represents the 13th most deadly cancer, and it is responsible for causing at least 100,000 deaths in 2018 (2.8% of all cancer deaths), with a mortality rate four times greater in men (3.2/100,000) compared with women (0.9/100,000) (1).

The main risk factor in BC is tobacco smoke, which accounts for almost half of all urothelial BCs, followed by occupational exposure to aromatic amines and polycyclic aromatic hydrocarbons (2). Other minor risk factors are environmental pollution, diet, and genetic predisposition (3).

Most BC is only diagnosed following an episode of macroscopic haematuria. For this reason, successful treatment is closely related to early diagnosis, tailored therapy, and proper follow-up (2). Currently, cystoscopy combined with cytology is routinely used for diagnosis, prognosis, and disease surveillance (4).

BC can develop *via* two distinct entities: non-muscle-invasive BC (NMIBC) and muscle-invasive BC (MIBC). In NMIBC, the gold standard treatment contemplates a complete transurethral resection of bladder tumour and the subsequent induction and maintenance cycles with intravesical mitomycin chemotherapy or intravesical Bacille Calmette–Guerin (BCG) immunotherapy (TURBT) (5). MIBC requires, instead, a multimodal approach to achieve the best chance in terms of prognosis, which comprises different treatments such as neoadjuvant chemotherapy and radical cystectomy with an open, laparoscopic, or robotic approach (6). It is although possible, in selected MIBC patients, to offer a bladder-sparing alternative, which includes, similarly to NMIBC, transurethral resection followed by chemotherapy and/or radiotherapy (5).

Clinical factors such as stage, tumour grade, presence of carcinoma *in situ* (CIS), age, and gender are well-known predictors for progression to MIBC, whereas multiplicity, tumour size, and prior recurrence are the most important predictors for recurrence (7).

In recent years, the role of the immune system has been widely studied in its involvement in tumorigenesis, cancer progression, and prognosis (8). Lymphocytes, in particular, seems to play an important role in suppressing cancer cell proliferation and migration (9); and similarly, the role of tumour-infiltrating lymphocytes (TILs) has been validated as a fundamental counterbalance in tumour immune environment (9–11). Analogously, monocytes have also an essential role in tumorigenesis. Indeed, they differentiate into tumour-associated macrophages (TAMs), which are attracted to tumour tissue by chemotactic factors and are representative of tumoural burden, in addition to being associated the outcomes of various cancers. As monocyte percentage could be used as a reflection of TAMs, lymphocyte percentage could be considered as the expression of the equilibrium between antitumor immune reaction and tumour promotion (12).

As result, novel haematological markers have been identified as independent predictors of cancer progression, counting platelet-to-lymphocyte ratio (PLR), neutrophil-to-lymphocyte ratio (NLR), and lymphocyte-to-monocyte ratio (LMR) among the most used (12, 13). Several studies have already reported their successful use in a variety of human cancers such as hepatocellular carcinoma, colorectal cancer, and cholangiocarcinoma (14–16).

A meta-analysis conducted by Nishijima et al., underlining the importance of the immune system in cancer pathogenesis, analysed LMR as a prognostic factor in various cancers. In different outcomes observed [overall survival (OS), cancer-specific survival (CSS), and disease-free survival (DFS)], a low pre-treatment LMR represented an unfavourable and robust prognostic factor in patients with non-haematological cancers (8).

Regarding BC, most studies concerning the link between the LMR ratio and this cancer are focused on MIBC patients undergoing radical cystectomy. In contrast, little information is available in literature regarding NMIBC patients undergoing BCG immunotherapy.

Yoshida et al. and Temraz et al. were among the first to highlight the significant positive correlation between high LMR and improved OS and time to recurrence (TTR) in MIBC patients (17, 18). A recent meta-analysis by Ma et al. evaluated the prognostic value of pre-treatment LMR in MIBC accounting for OS, recurrence-free survival (RFS), and CSS. In nine studies considered (for a total of 5,638 BC patients), high LMR patients reported better OS (hazard ratio (HR) 0.63, 95%, p < 0.0019), better RFS (HR 0.59, 95%, p = 0.017), and better CSS (HR 0.76, 95%, p > 0.001). Conversely, a low LMR was associated with older age (>60), poor tumour differentiation, higher stages (3, 4), presence of lymph nodal metastases, or concomitant presence of CIS (12).

Regarding NMIBC, Adamkiewicz et al., in a retrospective analysis on 125 NMIBC patients undergoing BCG immunotherapy, evaluated, in a model comprehending stage, grade, age, gender, and smoking status, the best prognostic value among these novel markers. LMR reported the highest prognostic values [area under the curve (AUC) = 0.756] with a cut-off point of 3.25, outperforming NLR and PLR in terms of progression prediction. Adding LMR to the baseline indeed significantly increased the AUC by 0.08 (p = 0.001), while NLR and PLR did not increase AUCs significantly to the baseline model. Despite the significance of these pioneering results, the prognostic value of LMR has not previously studied in NMIBC patients, and the study did not incorporate in the model the possible effects of other co-morbidities or drugs, which could have altered biomarker values (19).

Nevertheless, due to these premises, and considering the morbidity and mortality of BC, an accurate prediction model of disease progression is critical and fundamental in cancer management in order to provide the best treatment option and, hopefully and potentially, improve clinical outcomes for NMIBC patients (20).

LMR could be considered as a valuable independent predictor of progression both in NMIBC patients receiving immunotherapy and in MIBC patients undergoing radical cystectomy. In addition, the performance of this marker, the easily availability, and the limited costs permit the consideration of LMR as a good prognostic biomarker. Overall, its addition in more complex prognostic models could quickly and simply increase the precision of the former.

Although the approval of LMR as a new prognostic biomarker in NMIBC and MIBC is the first step towards a definition of better predictive models of progression, further efforts are required to strengthen LMR as a new indicator of progression.

## Author Contributions

All authors listed have made a substantial, direct, and intellectual contribution to the work and approved the manuscript for publication.

## Conflict of Interest

The authors declare that the research was conducted in the absence of any commercial or financial relationships that could be construed as a potential conflict of interest.

## Publisher’s Note

All claims expressed in this article are solely those of the authors and do not necessarily represent those of their affiliated organizations, or those of the publisher, the editors and the reviewers. Any product that may be evaluated in this article, or claim that may be made by its manufacturer, is not guaranteed or endorsed by the publisher.
